# Polysaccharides from *Chrysanthemum morifolium* Ramat ameliorate colitis rats by modulating the intestinal microbiota community

**DOI:** 10.18632/oncotarget.20477

**Published:** 2017-08-24

**Authors:** Jin-Hua Tao, Jin-Ao Duan, Shu Jiang, Nan-Nan Feng, Wen-Qian Qiu, Yong Ling

**Affiliations:** ^1^ School of Pharmacy, Nantong University, Nantong 226001, PR China; ^2^ Jiangsu Collaborative Innovation Center of Chinese Medicinal Resources Industrialization, Nanjing University of Chinese Medicine, Nanjing 210023, PR China

**Keywords:** chrysanthemum polysaccharides, ulcerative colitis, 16S rRNA, microbial diversity, short chain fatty acids

## Abstract

The gut microflora dysbiosis has been closely related with the inflammatory bowel disease (IBD). In this study, the effect of polysaccharides from *Chrysanthemum morifolium* Ramat on the gut microbiota was evaluated by ulcerative colitis (UC) rat model. Physiological and pathological analyses suggested that Chrysanthemum polysaccharides possessed notably protective effects on UC *in vivo*. Based on the Illumina MiSeq platform, 16S rRNA sequencing of the rat colonic contents indicated that the intestinal flora structure remarkably changed in the model rats and the tendency was alleviated to a certain degree by treatment with different dosages of Chrysanthemum polysaccharides. In normal groups, there were more *Firmicutes* than *Bacteroidetes*, but this change lost at the pathological state. Following Chrysanthemum polysaccharides, rising *Firmicutes*/*Bacteroidetes* ratio was validated. Besides the microbial diversity and the community richness of the UC rats were improved by Chrysanthemum polysaccharides, the composition of intestinal microflora in the model group were also restored after oral administration of Chrysanthemum polysaccharides. The abundance of opportunistic pathogens was decreased (*Escherichia, Enterococcus* and *Prevotella*), while the levels of protective bacteria such as *Butyricicoccus* and *Clostridium* (butyrate-producing bacteria), *Lactobacillus* and *Bifidobacterium* (probiotics), *Lachnospiraceae* and *Rikenellaceae* elevated in various degrees. Correlation analysis between intestinal flora and biochemical factors suggested that the relative abundance of protective bacteria was positively correlated with the levels of anti-inflammatory cytokines such as IL-4, IL-10 and IL-11, while aggressive bacteria were positively correlated with proinflammatory cytokine such as IL-23、IL-6、 IF-17、TNF-α、IL-1β and IFN-γ. The above results showed that the intestinal flora were closely related to the secretion and expression of cytokines in the body, and they interacted with each other to regulate immune function. Thus, Chrysanthemum polysaccharides could ameliorate ulcerative colitis by fostering beneficial intestinal flora growth, modulating the balance of intestinal microecology and restoring the immune system.

## INTRODUCTION

Inflammatory bowel disease (IBD) encompasses two clinical forms such as ulcerative colitis (UC) and Crohn's disease (CD), which possesses some symptoms such as weight loss, severe diarrhea and abdominal pain, seriously impairing the quality of life [1, 2]. To date, the pathogenesis of IBD remains unclear, though several factors have been associated with disease development including immune dysregulation, genetics, risk alleles, barrier dysfunction, environmental exposures and, more recently, compositional changes in the communities of bacteria that inhabit the gut [3–5]. The intestinal microflora dysbiosis is closely related to the gastrointestinal disease IBD [6]. The intestinal microflora, approximately 100 trillion microorganisms, is a large bacterial community that colonizes in the intestine, with powerfully metabolic activities for various enzymes and other functions such as intestinal epithelial cell proliferation/differentiation, intestinal development and barrier function that affect the physiology and pathology of the host's mucosal immune system [7–9]. Many researches have overwhelmingly verified the roles of bacteria in IBD. Chronic intestinal inflammation in transgenic and gene knock-out rats occurs under standard laboratory conditions, but not in a germ-free environment [10, 11].

Nowadays numerous researches have focused on profiling the microbial diversities in IBD and their differences with healthy individuals, which show that bacterial diversity, stability and cluster in IBD have been notably reduced. Based on direct sequencing of variable bands and verification by real time PCR, the intestinal bacteria such as *Bacteroides, Eubacterium*, and *Lactobacillus* were reduced in IBD [12]. Using metagenomic approach, Manichanh *et al* detected a reduced complexity of the bacterial phylum *Firmicutes* as a signature of the faecal microbiota in patients with IBD [13]. *Bacteroidetes* and *Proteobacteria* significantly increased in CD patients, while *Clostridia* reduced [14]. *Firmicutes* decreased in IBD along with increase of *Bacteroidetes* [2]. Some of the recent studies have also indicated the crucial role of *Proteobacteria* in the pathogenesis of ulcerative colitis [15, 16]. Additionally, intestinal bacteria help supply host nutrition by producing short chain fatty acids (SCFAs) and vitamins, and play crucial roles in the maintenance of human health by preventing pathogen colonization and by shaping and maintaining normal mucosal immunity. SCFAs have established anti-inflammatory effects in a variety of animal colitis models [17].

Chrysanthemum, the dry capitulum of *Chrysanthemum morifolium* Ramat, has been clinically used to effectively cure some infectious and inflammatory diseases such as influenza, colitis, stomatitis for thousands of years in Korea and China. Additionally, it has been also applied to treat cancer, sores, vertigo and hypertension [18–21]. Our previous studies have demonstrated that the polysaccharides from *Chrysanthemum morifolium* Ramat possessed remarkably protective effects on DSS induced colitis by increasing SCFAs production [21]. However, the mechanisms on the colonic fermentation were deficiency and also limited data exist on the impact of Chrysanthemum polysaccharides on the gut microbiota.

Recently, based on the next generation sequencing Illumina Miseq of 16S rRNA gene libraries driven by high-throughput technologies, characterization of bacterial community diversity has been possible [22]. Assembly of short sequences into operational taxonomic units (OTUs) is an initially key process in analyzing metagenomic data [23]. In this study, the above method was used to evaluate dynamic changes of intestinal microflora in 2, 4, 6-trinitrobenzene sulfonic acid (TNBS) induced colitis after oral administration of polysaccharides from *Chrysanthemum morifolium* Ramat. Meanwhile, the correlation analysis between intestinal flora and colitis-related biochemical factors was carried out to explore the possible mechanism of chrysanthemum polysaccharides to ameliorate TNBS-induced colitis. The Supplementary Figure 1 showed the experimental strategy.

## RESULTS

### Amelioration of Chrysanthemum polysaccharides on TNBS-induced colitis

**Figure 1:**
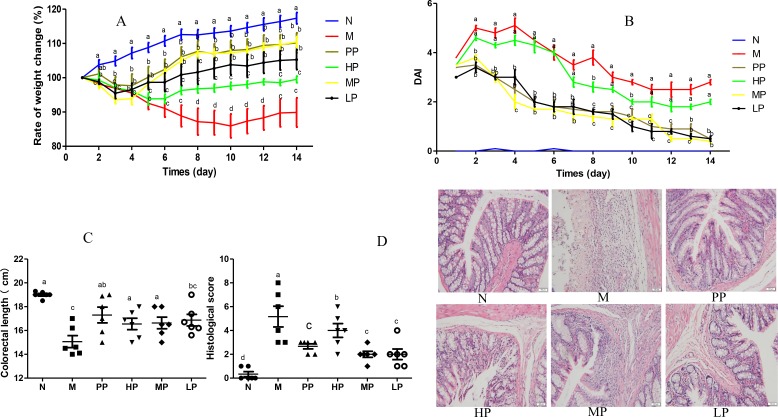
Chrysanthemum polysaccharides ameliorated TNBS-induced colitis in SD rats **(A)** Change of body weight during the disease process. **(B)** DAI based on weight loss, hematochezia, and diarrhea. **(C)** Statistics of colon length of each group. **(D)** Histopathological changes of colons. Significant differences (P < 0.05) between treatments were indicated by the letters a, b, or c. The results were presented as the mean ± SD; n = 6 for each treatment. N, normal group; M, TNBS-induced group; PP, SASP 0.5g/kg; HP, 200 mg/kg; MP, 100 mg/kg; LP, 50 mg/kg.

In this study, intra-colonic instillation of TNBS-induced colitis rat model was established and successfully applied to evaluate the amelioration of Chrysanthemum polysaccharides. From the fourth day of rectal administration of TNBS, rats showed increasingly severe symptoms such as serious diarrhea, obvious rectal bleeding and notable body weight loss. Compared to the normal group (Figure 1A), TNBS-induced colitis rats (M) remarkably lost weight throughout the trial period (p < 0.01), which was rescued by the Chrysanthemum polysaccharides treatment (HP 200 mg/kg, MP 100 mg/kg, LP 50 mg/kg). Disease activity index (DAI) was prominently higher in the model group than that in the normal group (p < 0.01). Comparison with the model group, treatments with low and middle doses of Chrysanthemum polysaccharides and SASP notably reduced DAI (p < 0.01). However, no remarkable differences were observed in HP groups (Figure 1B). Shortened colon length is an important physiological index of colitis. TNBS treated rats showed substantial reduction in colon length compared to the normal group (p < 0.001). Chrysanthemum polysaccharides at 50 mg/kg alleviated the situation of colon shortening (Figure 1C) (p < 0.05). The histopathological characteristics of colon tissues from each group were evaluated by H&E staining. The results indicated that severe pathological changes such as mucosal lesion, necrosis and infiltration of inflammatory cells including monocytes and neutrophils occurred in the colonic tissues of model rats, which were alleviated by Chrysanthemum polysaccharides treatment, especially the MP (100 mg/kg) and LP (50 mg/kg) (p < 0.01) (Figure 1D).

**Figure 2:**
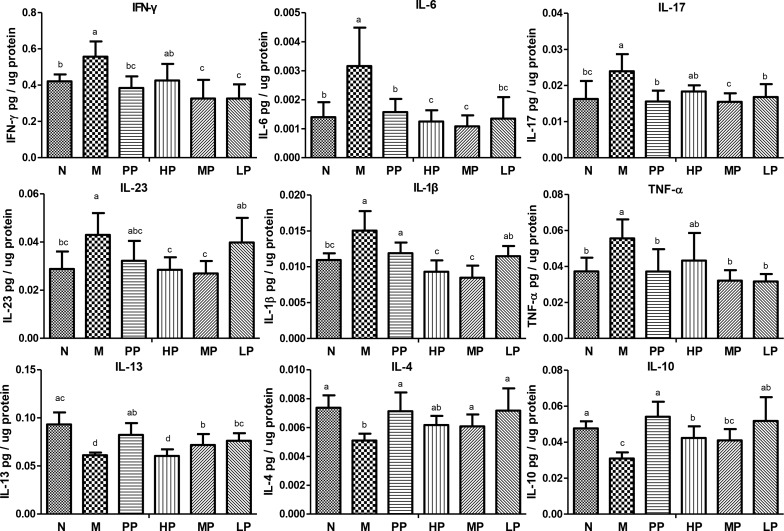
Chrysanthemum polysaccharides regulated cytokine profiles in colons mucosa of SD rats with TNBS-induced colitis Cytokine levels in homogenated colonic proteins were assessed by ELISA. Significant differences (P < 0.05) between treatments were indicated by the letters a, b, or c. The results were presented as the mean ± SD; n = 6 for each treatment. N, normal group; M, TNBS-induced group; PP, SASP 0.5g/kg; HP, 200 mg/kg; MP, 100 mg/kg; LP, 50 mg/kg.

To further evaluate the association between the alleviation of TNBS-induced colitis and inflammatory factors/cytokines in colitis rats, the levels of these factors/cytokines in colon epithelial tissue samples were quantified. Compared with normal rats, the levels of many cytokines including IL-6, TNF-α, IL-17, IL-23, IL-1βand IFN-γ were increased, while IL-13, IL-10 and IL-4 were decreased in TNBS-induced colitis rats. However, the cytokine levels tended to be restored to the normal group after oral administration of Chrysanthemum polysaccharides (Figure 2).

### High-throughput 16S rRNA sequencing of the rat colonic contents

In this study, 30 gut content samples were applied to evaluate the amelioration of Chrysanthemum polysaccharides on TNBS-induced colitis. A high throughput sequencing on the Illumina MiSeq platform was performed and the results showed a total of 1,086,952 reads, which passed all quality filters under 97% identity conditions to obtain a total of 10,653 species classification OTUs. Each sample was covered by an average of 36,231 reads (Table 1). The individual rarefaction curves and Shannon Wiener curves of samples were close to the saturation plateau (Supplementary Figure 2), revealing that high sampling coverage (∼99%) was obtained in all samples.

**Table 1:** Richness and diversity estimation for colon contents bacterial populations based on alpha diversity analysis

group	0.97			
	ACE	Chao1	Shannon	Simpson
N	420.2±13.1	421.2±12.8	3.752±0.09	0.0599±0.008
M	367.4±36.8*	339.6±19.8*	3.104±0.29*	0.0634±0.015
PP	359.6±41.4	379.4±52.9	3.684±0.22	0.0707±0.024
HP	404.2±40.9	407.8±46.3	3.658±0.53	0.0902±0.055
MP	417.0±39.0	423.8±43.5^#^	3.958±0.32^#^	0.0530±0.011
LP	453.2±23.2^#^	458.8±27.3^##^	3.940±0.32^#^	0.0535±0.032

*, P<0.05, M *VS* N; ##, P<0.01, #, P<0.05, polysaccharides group *VS* M.

The ACE and Chao1 reflect the community richness of species within a single sample, while Shannon and Simpson indexes represent microbial diversity. In Table 1, the ACE, Chao1, Shannon and Simpson indexes in the TNBS group were much lower than those in the normal control group, which were dramatically risen by Chrysanthemum polysaccharides treatment (p<0.01). However, statistical differences in alpha diversity were not observed among the three dosage (HP 200 mg/kg; MP 100 mg/kg; LP 50 mg/kg) of Chrysanthemum polysaccharides (P > 0.05).

### OTU network analyses of bacterial communities

**Figure 3:**
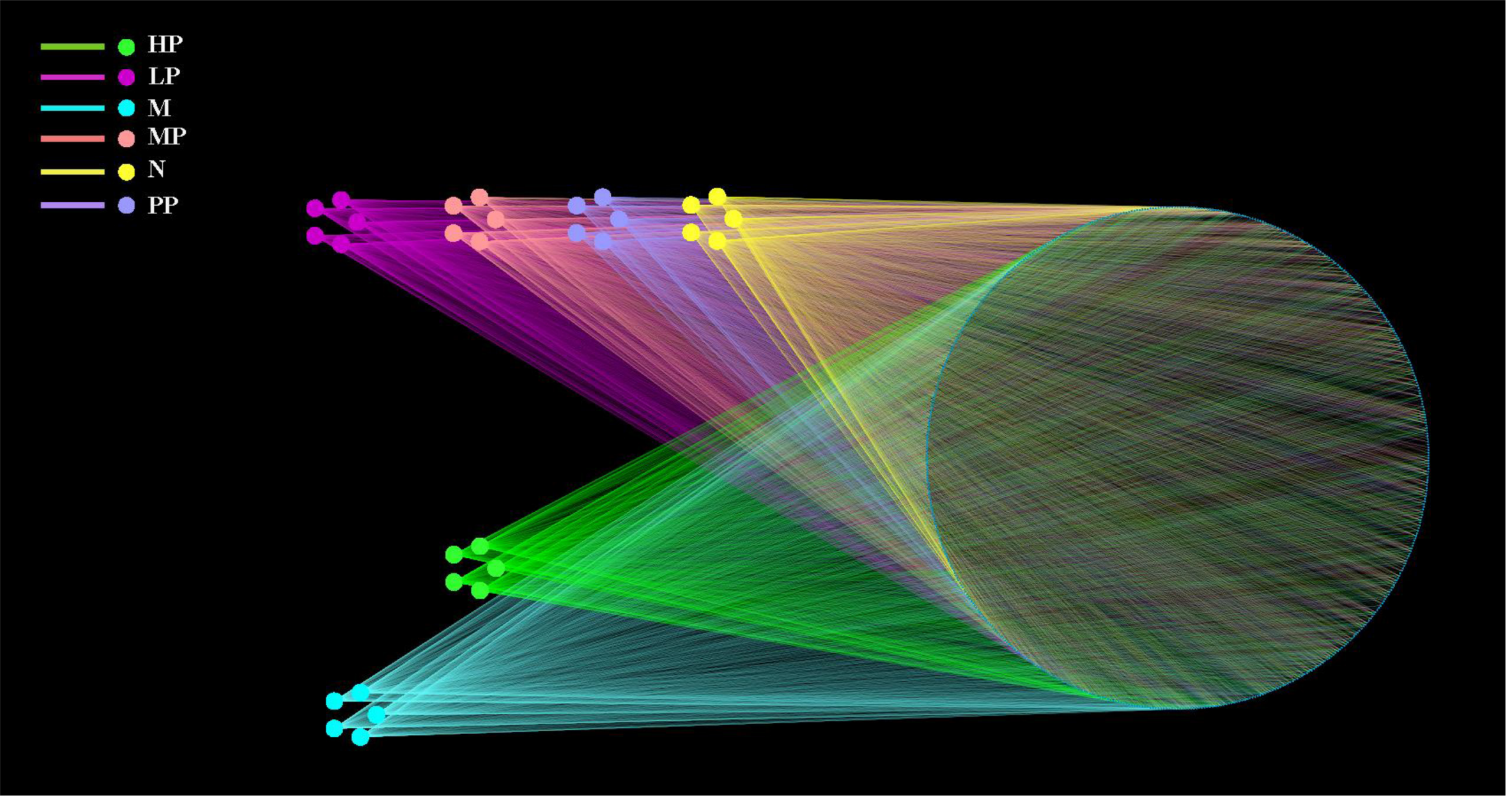
OTU network OTU network analysis of bacterial communities from different treatment groups (N, M, PP, HP, MP, LP) for the V3-V4 16S rRNA region.

OTUs and different treatment groups of (N, M, PP, HP, MP and LP) rats were labeled as nodes in bipartite network. OTUs were linked with the samples, and their sequences would be found in OTU-nodes [24]. The OTUs network-based analyses showed that samples from LP, MP and PP were more closely related to N compared to HP and M (Figure 3), which suggested that samples among LP, MP and PP possessed higher similarity than that among HP and M. The above analyses would provide supports for the hypothesis that different treatment factors were selective pressures on microbiota and play key roles in the protective effects on TNBS-induced colitis.

### Comparison of the gut bacterial community among different treatment groups

**Figure 4:**
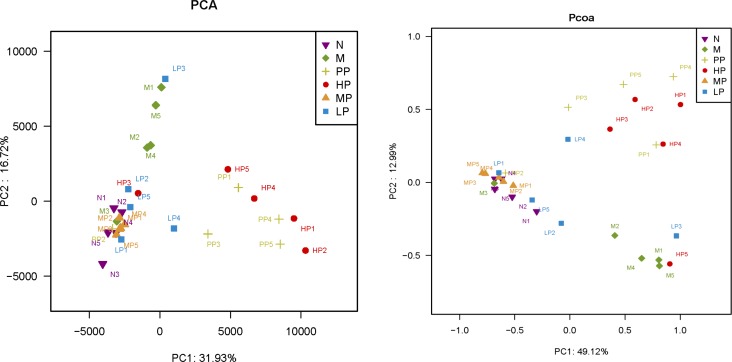
PCA (left) and PCoA (right) analysis of variation between the bacterial communities present in all biopsy samples Each data point represents an individual sample.

In this study, notable differences in richness and diversity of intestinal microflora among the different treatment groups were observed. A phylogenetic tree was used to detect changes of intestinal microflora among different treatment groups (Supplementary Figure 3). The greatest variations in intestinal microflora were found at the treatment of low dosage of Chrysanthemum polysaccharides (LP, 50 mg/kg), which approached the normal group. Minimal inter-rat variation was detected in other treatment groups except for a special one. Bacterial communities were also clustered by using two dimensional of PCA and PCoA of unweighted unifrac distance matrices. As was shown in Figure 4, there was notable separation between model and normal rats. Model rats treated with middle and low dosage of Chrysanthemum polysaccharides respectively (MP, 100 mg/kg; LP, 50 mg/kg) had higher similarities in bacterial members with normal rats through clustering closely on the two dimensional PCA and PCoA plot. The results revealed that Chrysanthemum polysaccharides had amelioration effect on TNBS-induced colitis.

Linear discriminant analysis (LDA) linked with effect size measurements was used to detect major bacteria in the different treatment groups. The major bacteria were multifarious in the N, M, PP, HP, MP and LP treatment groups (Supplementary Figure 4). *Ruminococcaceae, Prevotellaceae, Prevotella* 9, *Ruminococcaceae* UCG_005, *Blautia, Clostridiumsensustricto1, Clostridiaceae1, Faecalitalea, Ruminococcus1, Selenomonadles, Phascolarctobacterium, Acidaminococcaceae* and *Negativicutes* were the core microbes observed in the TNBS-induced colitis (M), while *Fiemicutes, Clostridiales, Clostridia, Bacilli, Lactobacillales, Lactobacillaceae, Lactobacillus, Romboutsia* and *Peptostreptococcaceae* were the core microbes in the normal group (N). The results were accordant with the previous report that *Clostridiaceae, Prevotellaceae* and *Ruminococcaceae* were observed to be at higher levels in the individuals suffering from chronic inflammatory bowel disease [16]. In the Chrysanthemum polysaccharides treatment groups, *Bacteroidetes, Bacteroidales, Bacteroidia, Bacteroidaceae, Bacteroides* and *Prevotellaceae* UCG_001 were the core microbes in HP; *Lachnospiraceae* NK4A136, *Ruminococcaceae* UCG_014, *Turicibacter* and *Enterorhabdus* were the core microbes in MP; and *Akkermansia, Verrucomicrobiae, Virrucomicrobia, Verrucomicrobiaceae, Verrucomicrobiales, Moryella, Erysipelatoclostridium, Enterococcaceae, Enterococcus, Coriobacteriaceae* UCG_002, *Ruminococcaceae* UCG_009, *Anaerostipes, Allobaculum, Catabacter, Bifidobacetriales, Bifidobacterium, Bifidobacteriaceae* and *Bacteroidetes* were the core microbes in LP.

### Screening of characteristic differential bacteria and intervention of Chrysanthemum polysaccharides

**Figure 5:**
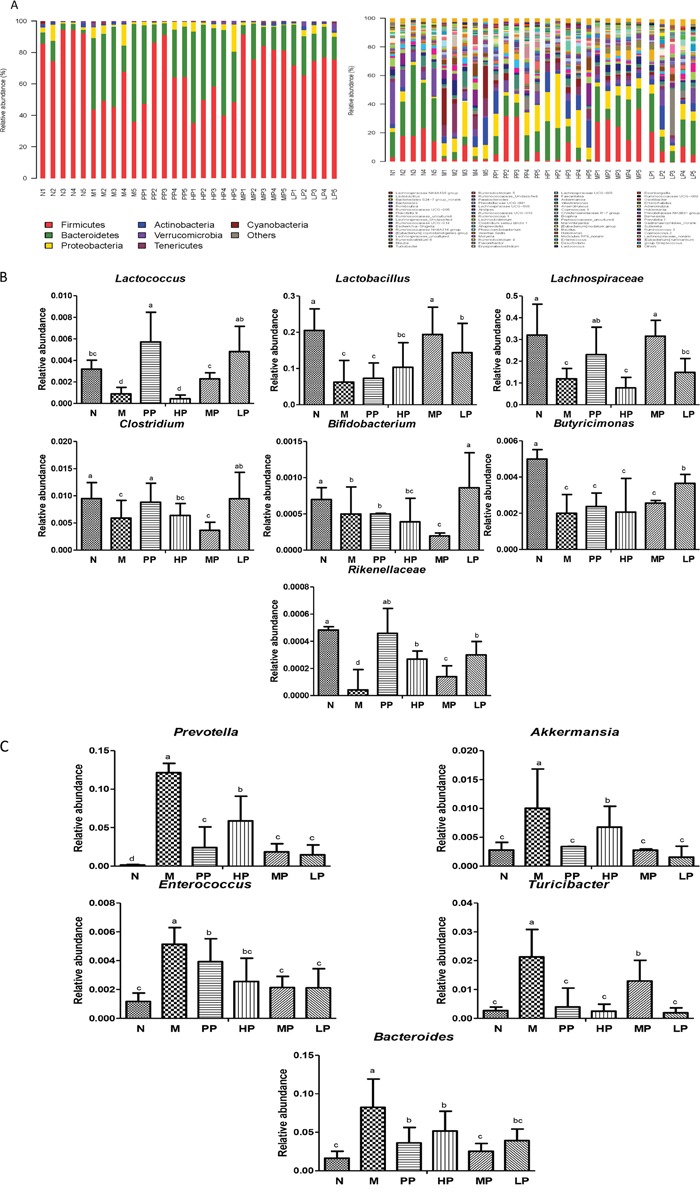
**(A)** Relative abundance of the main phyla and genes in the intestinal microbiota. Left: Phyla; Right: Genes. **(B)** Average relative abundance of seven protective bacteria in intestinal contents of different treatment groups. **(C)** Average relative abundance of five aggressive bacteria in intestinal contents of different treatment groups. Significant differences (P < 0.05) between treatments were indicated by the letters a, b, or c. The results were presented as the mean ± SD; n = 5 for each treatment. N, normal group; M, TNBS-induced group; PP, SASP 0.5g/kg; HP, 200 mg/kg; MP, 100 mg/kg; LP, 50 mg/kg.

To corroborate the correlation between intestinal microflora and the effects of Chrysanthemum polysaccharides treatment, the abundance of different bacteria in all samples was analyzed. Totally 11 different bacterial phyla were identified. Composition and proportion of major bacteria largely shifted within the different treatment groups. The majorities of the sequences were *Bacteroidetes* (23.47%) and *Firmicutes* (70.88%), while the rest belonged to *Actinobacteria* (0.87%) and *Proteobacteria* (3.61%). In the 6 treatment groups, bacterial structures were similar, but composition proportion of different bacteria was quite different from each other. The same was true at the genus level (Figure 5A). Compositional comparisons revealed inconsistent results but have generally identified decrease of the *Firmicutes* phylum in IBD [2]. The ratio *Firmicutes*/*Bacteroidetes* was changed between the normal group and TNBS group (22.71±2.82 in N *vs* 2.73± 1.33 in M, P<0.001). In the normal group, there were much more *Firmicutes* than *Bacteroidetes*, but the difference lost at pathological state (Figure 5A). Following Chrysanthemum polysaccharides, rising *Firmicutes*/*Bacteroidetes* ratio was validated in the MP (8.11± 2.71) and LP (4.19± 1.30) groups, especially in the MP groups (P<0.01). Meanwhile, as were shown in Figure 5B and 5C, the relative abundance of twelve different flora, seven protective and five aggressive bacteria, in different treatment groups were addressed. For example, the facultative bacteria such as *Lactobacillus* and *Lactobacillaceae* were enriched in the normal group (20.51%), MP (19.38%) and LP (14.38%) respectively, while significantly decreased in TNBS-induced colitis (M) (6.24% *vs* 20.51%, P<0.01). What's more, *Lactobacillus* has been reported to own immunoregulatory properties [25]. After treatment of Chrysanthemum polysaccharides, the abundance of *Lachnospiraceae* (31.57% in MP *vs* 11.94% in M, P<0.001) and *Rikenellaceae* (0.026% in HP *vs* 0.004% in M, P<0.001; 0.014% in MP *vs* 0.004% in M, P<0.01; 0.029% in LP *vs* 0.004% in M, P<0.001) significantly increased, while the abundance of *prevotella* decreased (5.87% in HP *vs* 12.14% in M, P<0.05; 1.86% in MP *vs* 12.14% in M, P<0.001; 1.46% in LP *vs* 12.14% in M, P<0.001).

The commensal gut microbiota are very beneficial to the host, including maintenance of immune homeostasis [26], regulation of intestinal development, and enhancement of metabolic capabilities [27]. SCFAs, which are yielded majorly by bacterial metabolism, are important energy sources of colonic epithelial cells and can enhance the integrity of epithelial barrier and activate the gastrointestinal immune response, especially the butyrate [28–30]. The increase of butyrate-producing bacteria (e.g. *Butyricicoccus*, 0.37% in LP *vs* 0.20% in M, P<0.01; *Clostridium*, 0.95% in LP *vs* 0.59% in M, P<0.01) after oral administration of Chrysanthemum polysaccharides suggested that Chrysanthemum polysaccharides (LP, 50 mg/kg) had the ability to ameliorate TNBS-induced colitis by modulating the intestinal microbiota community (Supplementary Figure 4). Consistent with previous studies [31], the *Enterococcus* significantly increased in the TNBS-induced colitics group (0.51% in M *vs* 0.12% in N, P<0.001) which decreased by Chrysanthemum polysaccharides. The abundance of *Bifidobacterium* was significantly increased by low dosage of Chrysanthemum polysaccharides (0.086% in LP *vs* 0.0.06% in M, P<0.01). Various *Bifidobacterium* strains (*breve, catenulatum, longum and infantis*) resulted in amelioration of intestinal inflammation in DSS-induced colitis in mice [32, 33].

**Figure 6:**
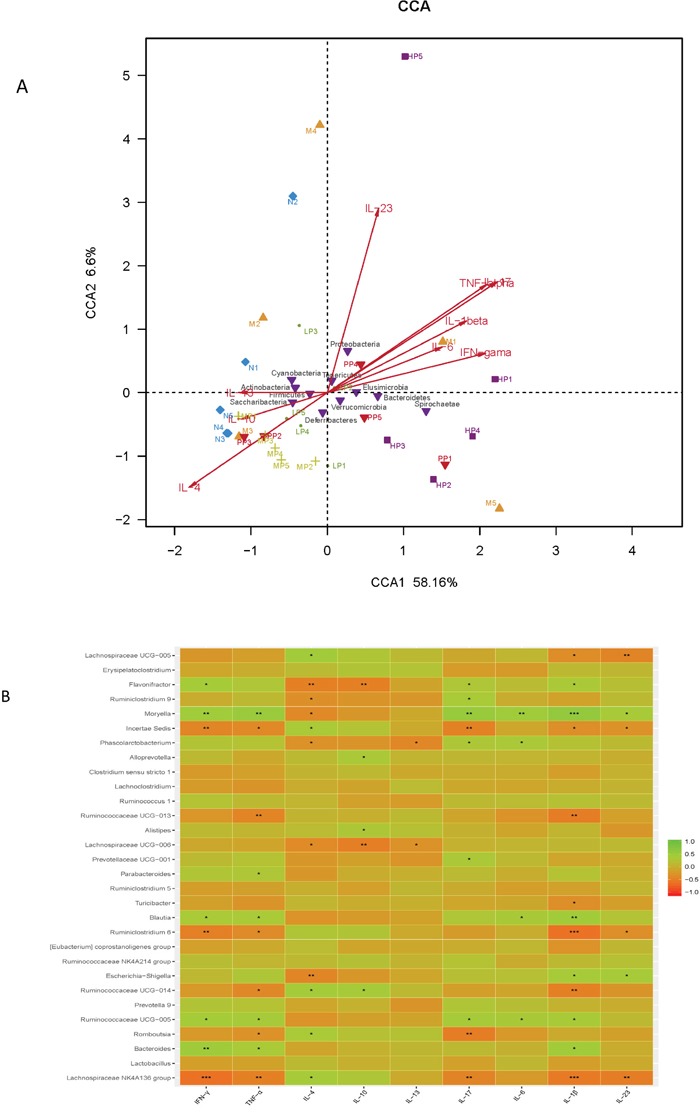
**(A)** The relationship between inflammatory factors/cytokines in colon epithelial tissue samples and intestinal flora based on CCA. **(B)** Heatmap correlation analysis of intestinal microflora and biochemical factors.

Multivariate direct gradients (CCA) were used to analyze the relationship between inflammatory factors/cytokines in colon epithelial tissue samples and intestinal flora. (Figure 6A and 6B). The results demonstrated that at phylum level, *Bacteroidetes, Proteobacteria* and *Elusimicrobia* (especially *Bacteroidetes)* were positively correlated with IL-23, TNF-α, IL-1β, IL-6, IF-17 and IFN-γ, which could promote the occurrence of inflammatory diseases. However, *Cyanobacteria, Firmicutes, Actinobacteria and Sacchribacteria* (especially *Firmicutes)* were positively correlated with IL-10, IL-13 and IL-4, which had the inhibition of inflammatory diseases. Obviously, both the MP and LP groups had positive effects on *Firmicutes*. At genus level, there was a significant positive correlation between protective bacteria such as *Butyricicoccus, Clostridium, Lachnospiraceae, Rikenellaceae, Lactobacillus* and *Bifidobacterium* and anti-inflammatory cytokine such as IL-4、IL-10、IL-11, while aggressive bacteria such as *Prevotella, Ruminococcus, Bacteroides, Escherichia-Shigella, akkermansia, Turicibacter* were positively correlated with proinflammatory cytokine such as IL-23、TNF-α、IL-1β、IL-6、IF-17 and IFN-γ.

The above results showed that the intestinal flora were closely related to the secretion and expression of cytokines in the body, and they interacted with each other to regulate immune function. Chrysanthemum polysaccharides could ameliorate TNBS-induced colitis by fostering beneficial intestinal flora growth, modulating the balance of intestinal microecology and restoring the immune system.

## DISCUSSION

The intestinal microflora is recorded as the highest cell density for any ecosystem, which has a profound and crucial influence on human health, and changes of intestinal microbiome may be associated with bowel diseases [27, 34]. 16S rDNA sequence-based methods showed that two kinds of bacteria, *Bacteroidetes* and *Firmicutes*, were the major gut microflora constituting over 90% of the known phylogenetic categories [35]. Colitis is mainly characterized by tissue damage and colon inflammation. The intestinal microflora is crucial for protection of intestinal mucosa and microbiota dysbiosis could lead to mucosal injury [36, 37]. It is reported that faecal bacteria from healthy donors are expected to have therapeutic effects on patients with IBD [38]. In this study, disequilibrium in the microbiota was observed in the TNBS-induces colitis rats. Intervention with Chrysanthemum polysaccharides alleviated the imbalances, reduced inflammation, and relieved symptoms of disease. Using 16S rRNA gene sequences analysis of content samples from the normal group, TNBS-induced colitis and sequential Chrysanthemum polysaccharides treatment groups, we observed that profiles of predominant bacteria changed persistently. The ratio *Firmicutes*/*Bacteroidetes* was decreased while the increase was validated after Chrysanthemum polysaccharides treatment, especially in the MP group.

The SCFAs not only provide large amounts of energy for colonocytes but also possess anti-inflammatory characteristics, including the capability to decrease cytokine response induced by lipopolysaccharide [29, 39]. Thus, the lack of *Butyricicoccus* and *Clostridium* could make some individuals susceptible to gut inflammation.Additionally, decreased relative abundance of potentially immunomodulatory mucosal-associated species such as *Faecalibacterium prausnitzii, Clostridium leptum* and *Clostridium coccoides* have been reported for UC patient samples [40, 41]. These organisms could produce SCFAs which have established anti-inflammatory effects in a variety of animal colitis models [17, 29]. So, loss of SCFAs-producing bacteria from IBD patients is believed to represent at least one mechanism by which microbial dysbiosis contributes to the heightened immune activation associated with the disease. However, in our studies, the abundance of *Butyricicoccus* and *Clostridium* notably increased after Chrysanthemum polysaccharides treatment compared with the model group. The result suggested that Chrysanthemum polysaccharides (LP, 50 mg/kg) had the ability to ameliorate TNBS-induced colitis by modulating the intestinal microbiota community. Meanwhile, in our previous reports, compared with the DSS-treated colitis group, the SCFAs content in the different polysaccharides-treated group has remarkably increased which indicated that Chrysanthemum polysaccharides could contribute to bowel health by promoting SCFAs yield [21].

It is reported that enterotoxins can be produced by *Bacteroides* and *Clostridia* species characterized by proteolytic enzymes that heighten intestinal mucosal permeability and bacterial absorption [42]. In this study, more abundance of *Bacteroides* were observed in the model group than that in the normal group. However, the trend was reversed by Chrysanthemum polysaccharides (Figure 5C). Studies have revealed that virulence factors from adherent and invasive *E coli* are required for its colonization of intestinal mucosa, breakthrough of the epithelial barrier, interaction with host macrophages, and induction of proinflammatory cytokine synthesis [43–45]. The abundances of two kinds of opportunistic pathogens, *Escherichia* and *Enterococcus*, enriched in the TNBS group. However, the notable decrease of *Enterococcus* was observed in Chrysanthemum polysaccharides-treated groups, which could be correlative to the anti-inflammatory activities of Chrysanthemum polysaccharides relieving symptoms of disease by reducing some opportunistic pathogens. It was reported that, the strain *Lactobacillus* subspecies could reduce mucosal permeability, prevent colitis onset, and alleviate inflammatory reaction in IL-10-/- mice [37, 46]. *Lactobacillus* GG improved intestinal barrier function by inhibiting enterocyte apoptosis, and prevented colitis recurrence [47–49]. Previous studies have demonstrated that *Prevotella* was more abundant in IBD patients than in non-inflamed individuals. Consistent with the reports, we confirmed that the abundance of *Prevotella* increased heavily in the TNBS-induced colitics group, which decreased following Chrysanthemum polysaccharides treatment [2]. It indicated that the *Lachnospiraceae* could help maintain gastrointestinal tract health and be a powerful tool to evaluate intestinal health [50]. Alkadhi *et al.* found that *Rikenellaceae* abundance reduced in Muc2 deficient mice compared to healthy ones [51]. Thus, we have reason to believe that Chrysanthemum polysaccharides could prevent inflammatory disease by the changes of the gut microbial community.

It is believed that CD4 + T cells, which are an important part of the immune system, can not be neglected in the pathogenesis of IBD. The imbalance of maintenance-regulated factors/cytokines expression in Th1, Th2, Th17, Treg cells may be one of the main mechanisms of disease [52, 53]. In this study, following Chrysanthemum polysaccharides treatment, the levels of cytokines IFN-γ, IL-1β and TNF-α which secreted by Th1 were decreased significantly, so were IL-6, IL-17 and IL-23 which secreted by Th17, while IL-13, IL-10 and IL-4 which secreted by Th2 were increased. Unbalanced Th1/Th2, Th17/Treg was alleviated.

Correlation analysis between intestinal flora and biochemical factors indicated that there was a significantly positive correlation between protective bacteria such as *Butyricicoccus, Clostridium, Lachnospiraceae, Rikenellaceae, Lactobacillus* and *Bifidobacterium* and anti-inflammatory cytokines such as IL-4、IL-10 、IL-11, while aggressive bacteria such as *Prevotella, Ruminococcus, Bacteroides, Escherichia-Shigella, akkermansia, Turicibacter* were positively correlated with proinflammatory cytokines such as IL-23、TNF-α、IL-1β、IL-6、IF-17 and IFN-γ. The above results showed that the intestinal flora were closely related to the secretion and expression of cytokines in the body, and they interacted with each other to regulate immune function. Chrysanthemum polysaccharides foster beneficial intestinal flora growth, modulate the balance of intestinal microecology, restore the immune system, so as to ameliorate TNBS-induced colitis.

In conclusion, based on Illumina Miseq platform analysis, Chrysanthemum polysaccharides improved the microbial diversity and the community richness of the TNBS-induced rats, and the population composition and diversity of intestinal microflora in the TNBS group were restored after oral administration of Chrysanthemum polysaccharides. Opportunistic pathogens decreased (*Enterococcus, Escherichia* and *prevotella*), while the abundances of *Bifidobacterium, Butyricicoccus, Clostridium, Lachnospiraceae, Lactobacillus* and *Rikenellaceae* were elevated in various degrees. Thus, Chrysanthemum polysaccharides could ameliorate TNBS-induced colitis by modulating the intestinal microbiota community.

## MATERIALS AND METHODS

### Animals

Male Sprague-Dawley (SD) rats (body weight 220-250 g) were bought from the Vital River Laboratory Animal Technology Co., Ltd. (Beijing, China). The IACUC number is “SCXK2012-0001”. The animal experiments were performed according to the Regulations of Experimental Animal Administration (State Committee of Science and Technology of the People's Republic of China). The rats were housed in the specific pathogen-free (SPF) animal center for drug safety evaluation and research at Nanjing University of Chinese Medicine with an air-conditioned animal quarter with12 h light/12 h dark cycle at a temperature of 22 ± 2°C and a relative humidity of 50 ± 10%. All rats were acclimatized for 7 days before any experiments and were fed with standard chow and water ad libitum.

### Chemical regents

TNBS was bought from MP Biomedical (Aurora, OH, USA). Sulfasalazine (SASP) was obtained from Yi-feng Pharmacy in Nanjing, China. TNF-α, IFN-γ, IL-17, IL-6, IL-1β, IL-23, IL-13, IL-10 and IL-4 enzyme linked immunosorbent assay (ELISA) kits were obtained from Nanjing Jiancheng Bioengineering Institute Co., Ltd., Nanjing, China. The dried flowers of *C. morifolium* were purchased from Yang-ma town of She-yang county in Jiangsu province of China. Chrysanthemum polysaccharides were obtained according to our previous studies [21].

### Animal model of colitis

Acute colitis rats were induced by rectal administration of TNBS mixed with a certain percentage of ethanol through a special catheter [54]. Briefly, rats were anaesthetized with 10% chloral hydrate, and subsequently administered with 3 mL/kg of TNBS-ethanol solution (50 mg/mL) into the colon at 8 -10 cm depth from the rectum using a soft polyethylene catheter. The rats were fastened in the trendelenburg position for one minute to avoid loss of TNBS solution via the rectum. While normal rats were rectally administered with normal saline at equivalent instead of TNBS [55].

### Experimental procedures

24 hours (day 1) after induction of colitis, all the rats were randomly assigned to six groups, five rats were chosen in each group: Normal group (N), receiving normal saline at equivalent and received intragastric administration (ig) saline during treatment; TNBS model group (M), receiving ethanol vehicle with TNBS (TNBS + saline); Sulfasalazine group (PP), receiving SASP 0.5 g/kg (TNBS + SASP); Chrysanthemum polysaccharides (CP) high, middle and low dose treatment group (HP, MP, LP), receiving CP 200 mg/kg (TNBS + HP), 100 mg/kg (TNBS + MP) and 50 mg/kg (TNBS + LP), respectively. All above treatments from day 2 to day 15. The rats were detected daily for colitis by clinical symptoms including body weight, gross rectal bleeding and stool consistency, which was assessed by DAI according to the method described by Cooper [56]. 24 h after the last treatment (day 16), all rats were sacrificed after ether deep anesthesia. The colons were cut off, dissected along the longitudinal mesentery, rinsed with isotonic saline and subsequently used to assess colonic mucosa damage. Additionally, colonic contents were obtained. All the biological samples were stored at −80°C.

### Bacterial DNA extraction and PCR amplification

Microbial DNA was extracted from colonic contents with the E.Z.N.A.® Soil DNA Kit (Omega Bio-tek, Norcross, GA, U.S.). The V3-V4 regions of the bacterial 16S rDNA were amplified by PCR (95°C for 4 min, followed by 25 cycles at 95°C for 30 s, 55°C for 30 s, and 72°C for 45 s and a final extension at 72°C for 10 min) using primers 341F 5′-barcode- CCTAYGGGRBGCASCAG)-3′ and 806R 5′- GGACTACNNGGGTATCTAAT -3′. PCR reactions were carried out in triplicate 20 μL mixture including 5 μM primer (0.8 μL), 2.5 mM dNTPs (2 μL), FastPfu polymerase (0.4 μL), template DNA (10 ng) and 5×FastPfu buffer (4 μL). Amplicons were extracted from 2% agarose gels and purified with the AxyPrep DNA Gel Extraction Kit (Axygen Biosciences, Union City, CA, U.S.) and quantified with QuantiFluor™ -ST (Promega, U.S.).

### Illumina MiSeq sequencing

Purified PCR products were quantified by Qubit^®^3.0 (Life Invitrogen) and every twenty-four amplicons were mixed equally. The pooled DNA products were applied to establish Illumina Pair-End library by Illumina's genomic DNA library preparation procedure. Then the amplicon library was paired-end sequenced (2 × 250) on an Illumina MiSeq platform (Shanghai BIOZERON Co., Ltd) according to the standard protocols.

### Cytokine assay

Colonic tissue (50 mg) was extracted with 500 uL of guanidine HCl (5 M) and Tris-HCl (50 mM, pH 8.0) containing a protease inhibitor. Then, the extracts were centrifuged at 4°C (13, 000 g × 10 min), and subsequently the supernatant fractions were used to determine cytokines by ELISA kit (Nanjing Jiancheng Bioengineering Institute Co., Ltd. Nanjing, China) according to the manufacturer's instructions.

### Process of sequencing data

Raw fastq files were demultiplexed and quality-filtered with QIIME (version 1.17) [57, 58]. OTUs (97% similarity) were clustered with UPARSE version 7.1 http://drive5.com/uparse/) and chimeric sequences were authenticated and deleted by UCHIME. The genetic relationship of each 16S rDNA sequence was evaluated by Ribosomal Database Project Classifier (http://rdp.cme.msu.edu/) based on the silva database [59, 60].

### Diversity analysis

In the alpha diversity analysis which based on Mothur v.1.21.1 [61], community richness was estimated by the diversity indices including Chao 1 and ACE. Community evenness was assessed by the Simpson index and Shannon index. Estimators of population diversity, evenness and richness were calculated by OTUs (97% similarity). The beta diversity analysis was performed using UniFracto compare the results of the principal coordinate analysis (PCoA) and principal component analysis (PCA) [62].

### Statistical analysis

The experimental data were analyzed by GraphPad PrismV5 software. The results were expressed as mean ± standard deviation (X ± SD). The average numbers of groups were compared by one-way-ANOVA with Tukey's honestly significant difference (HSD) post hoc test. p<0.05 was considered to be remarkably different [63].

## SUPPLEMENTARY MATERIALS FIGURES



## Abbreviations

TNBS: 2, 4, 6-trinitrobenzene sulfonic acid
SASP: Sulfasalazine
N: Normal group
M: TNBS/ethanol model group
PP: TNBS/ethanol + SASP (0.5 g/kg)
HP: TNBS/ethanol + high dose treatment group (200 mg/kg)
MP: TNBS/ethanol + middle dose treatment group (100 mg/kg)
LP: TNBS/ethanol + low dose treatment group (50 mg/kg)
