# Supplementary material for: Polysaccharides from Chrysanthemum morifolium Ramat ameliorate colitis rats by modulating the intestinal microbiota community
Source: Oncotarget. 2017 Aug 24;8(46):80790–803. doi: 10.18632/oncotarget.20477 (PMC5655239; doi:10.18632/oncotarget.20477)
Supplement: Supplementary file 1 [file oncotarget-08-80790-s001.pdf]

Polysaccharides from *Chrysanthemum morifolium* Ramat ameliorate colitis rats by modulating the intestinal microbiota community

SUPPLEMENTARY MATERIALS

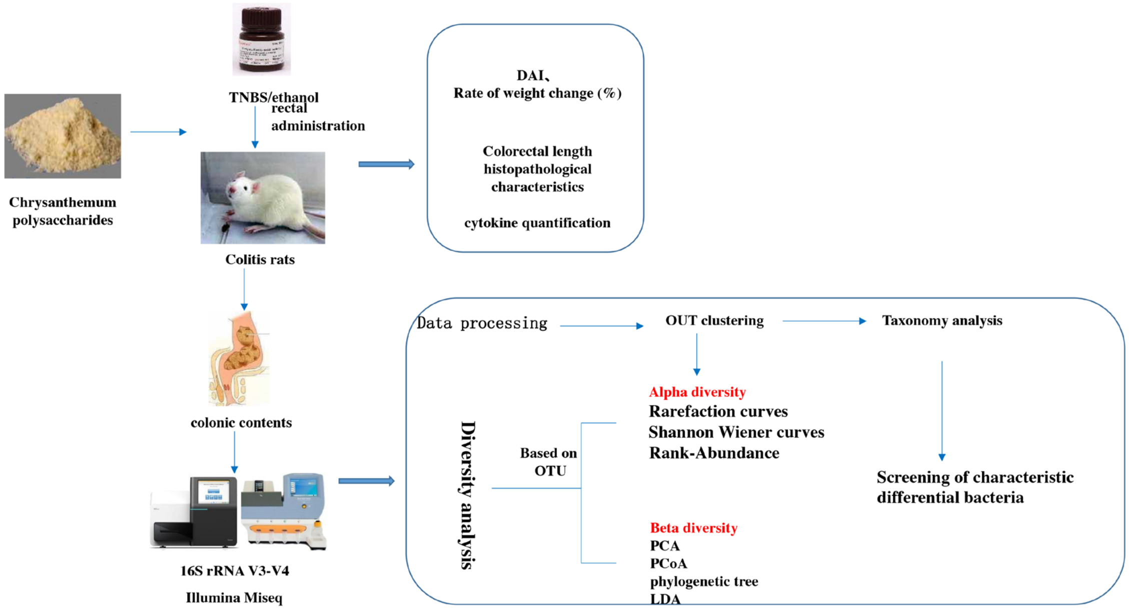

Supplementary Figure 1: The experimental strategy.

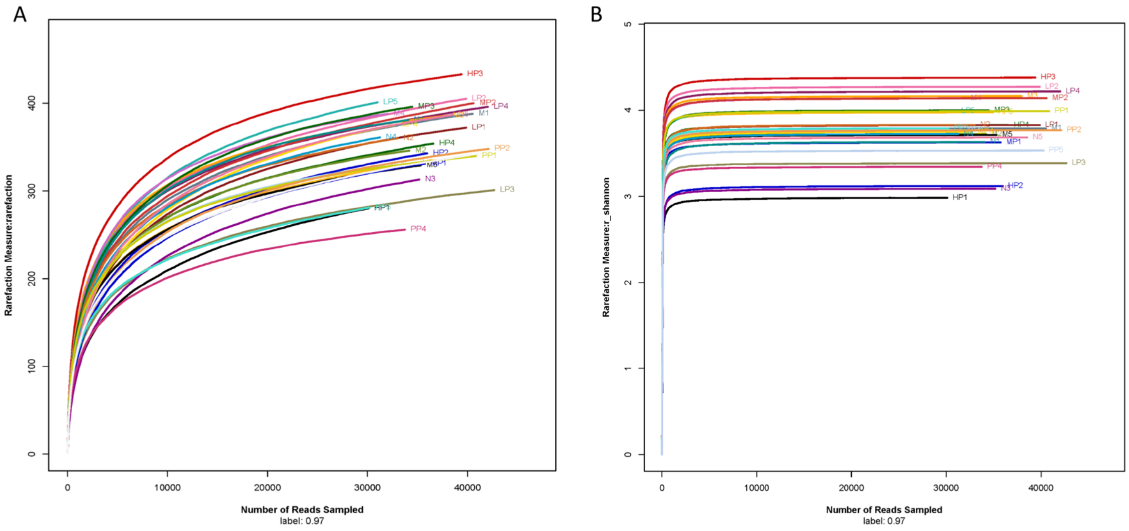

Supplementary Figure 2: (A) Individual rarefaction curves of OTUs clustered at 97% sequence identity. **(B)** Shannon Wiener curves of samples. N = 5 for each group.

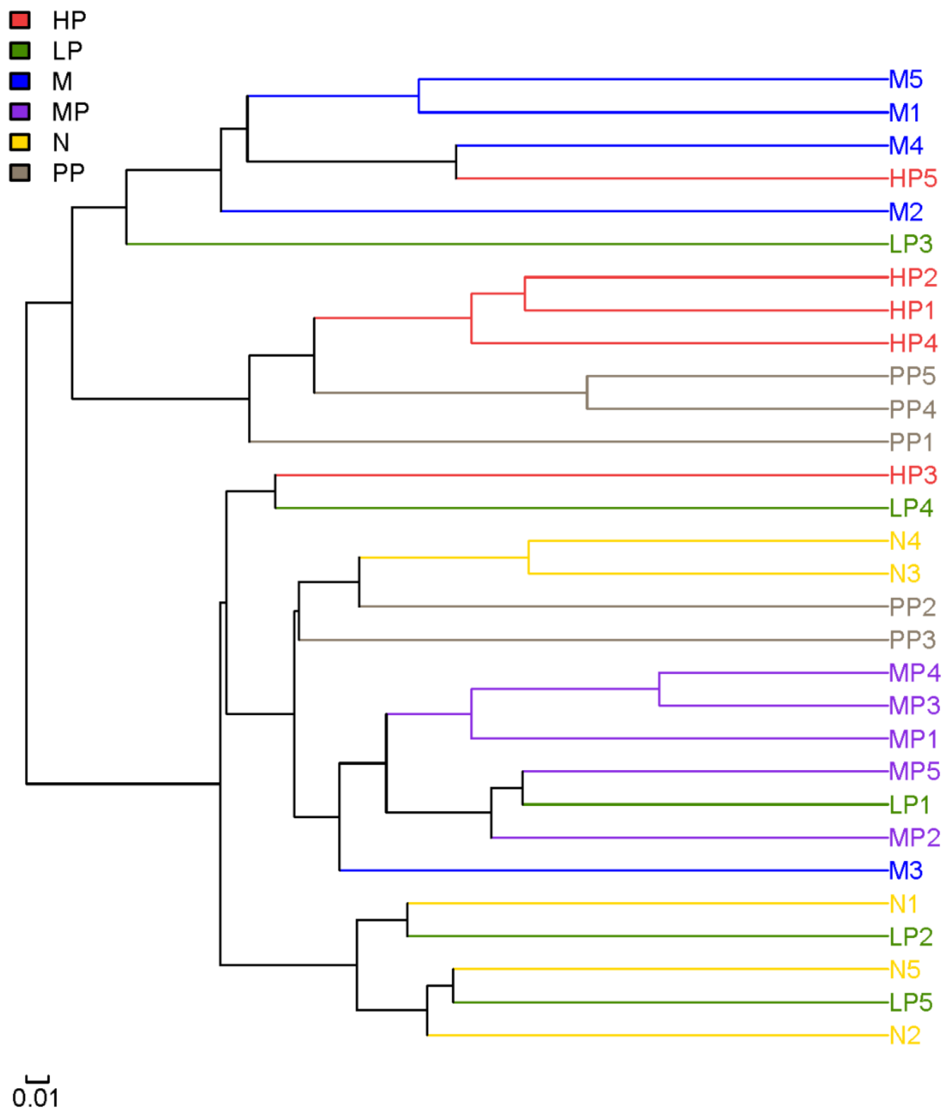

Supplementary Figure 3: **Multiple samples similarity tree.**

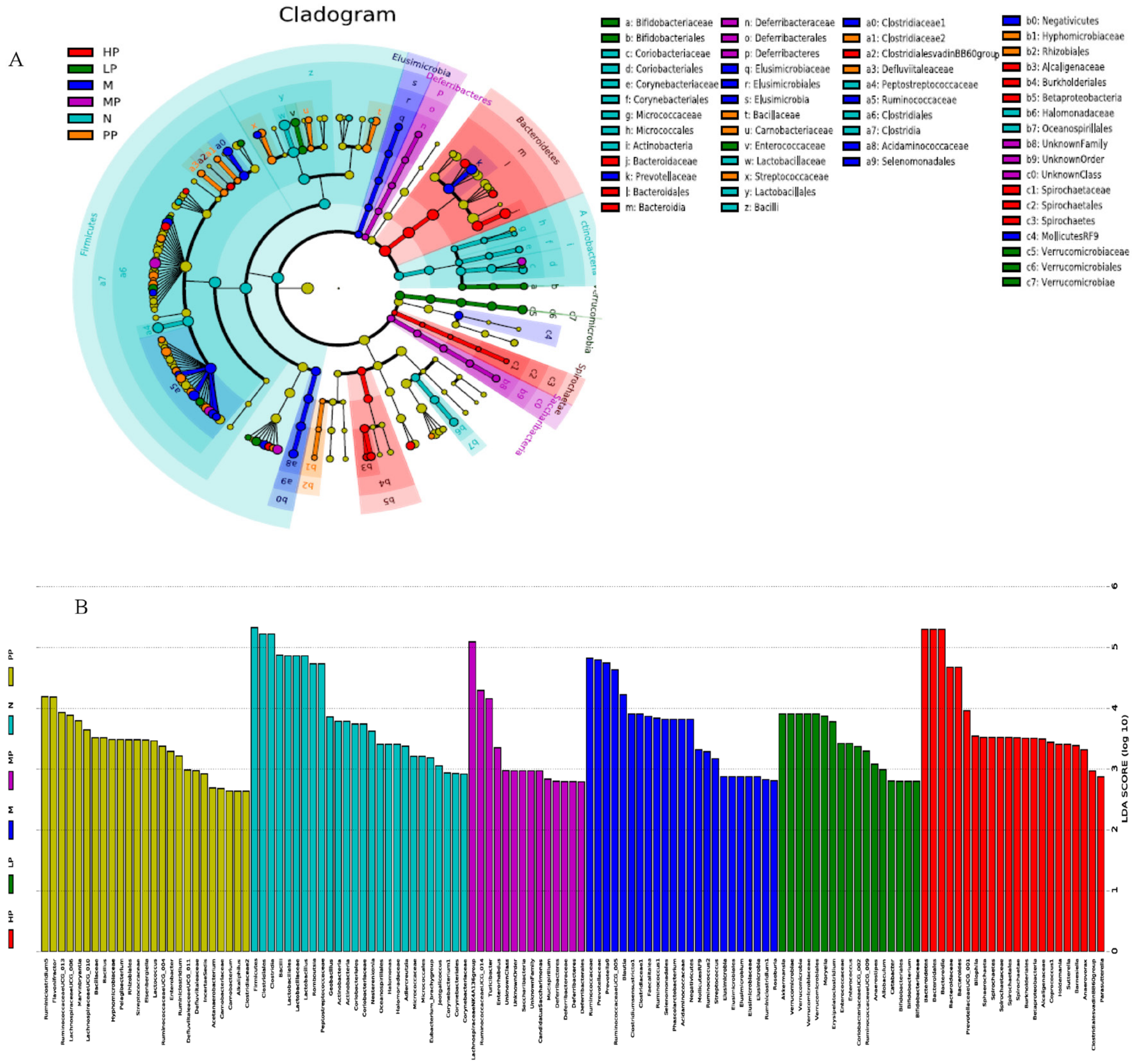

Supplementary Figure 4: LEfSE identified the most differently abundant taxon among the N, M, PP, HP, MP and LP group. (A) Cluster tree; **(B)** LDA scores of the taxa with significant effects.
